# Supplementary figures and images for: VE-cadherin junction dynamics in initial lymphatic vessels promotes lymph node metastasis
Source: Life Sci Alliance. 2023 Dec 26;7(3):e202302168. doi: 10.26508/lsa.202302168 (PMC10751244; doi:10.26508/lsa.202302168)

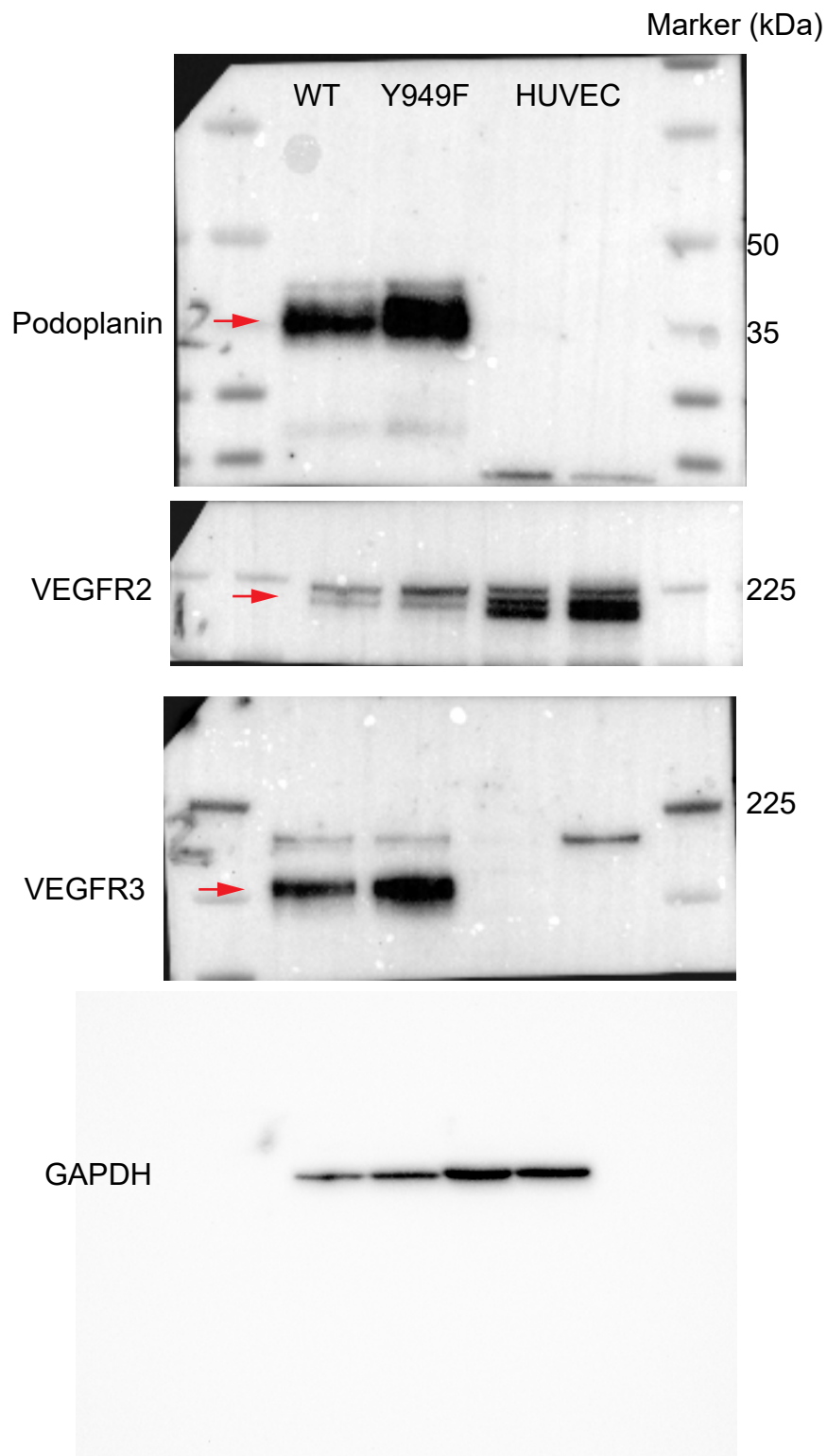

Supplement: Supplementary file 2 [file LSA-2023-02168_SdataF1.2.pdf]
